# Structural Rearrangement in an RsmA/CsrA Ortholog of *Pseudomonas aeruginosa* Creates a Dimeric RNA-Binding Protein, RsmN

**DOI:** 10.1016/j.str.2013.07.007

**Published:** 2013-09-03

**Authors:** Elizabeth R. Morris, Gareth Hall, Chan Li, Stephan Heeb, Rahul V. Kulkarni, Laura Lovelock, Hazel Silistre, Marco Messina, Miguel Cámara, Jonas Emsley, Paul Williams, Mark S. Searle

**Affiliations:** 1 School of Chemistry, Centre for Biomolecular Sciences, University of Nottingham, Nottingham NG7 2RD, UK; 2 School of Pharmacy, Centre for Biomolecular Sciences, University of Nottingham, Nottingham NG7 2RD, UK; 3 School of Life Sciences, Centre for Biomolecular Sciences, University of Nottingham, Nottingham NG7 2RD, UK; 4 Department of Physics, University of Massachusetts Boston, Boston, MA 02125, USA

## Abstract

In bacteria, the highly conserved RsmA/CsrA family of RNA-binding proteins functions as global posttranscriptional regulators acting on mRNA translation and stability. Through phenotypic complementation of an *rsmA* mutant in *Pseudomonas aeruginosa*, we discovered a family member, termed RsmN. Elucidation of the RsmN crystal structure and that of the complex with a hairpin from the sRNA, RsmZ, reveals a uniquely inserted α helix, which redirects the polypeptide chain to form a distinctly different protein fold to the domain-swapped dimeric structure of RsmA homologs. The overall β sheet structure required for RNA recognition is, however, preserved with compensatory sequence and structure differences, allowing the RsmN dimer to target binding motifs in both structured hairpin loops and flexible disordered RNAs. Phylogenetic analysis indicates that, although RsmN appears unique to *P. aeruginosa*, homologous proteins with the inserted α helix are more widespread and arose as a consequence of a gene duplication event.

## Highlights

• Phenotypic complementation identifies a CsrA/RsmA family member from *P. aeruginosa*
• Crystallography reveals a dimeric fold for RsmN
• The RsmN complex was solved with a hairpin motif from the noncoding sRNA RsmZ-2
• Details of binding affinity and specificity with target RNA 5′-ANGGAN motifs are revealed

## Teaser

The RsmA/CsrA family of bacterial RNA-binding proteins functions as global posttranscriptional regulators of mRNA. Morris et al. discover an additional family member in *P. aeruginosa*, RsmN. The structure of an RsmN/RNA complex reveals a unique protein fold and molecular details of base-specific RNA recognition.

## Introduction

The small RNA-binding proteins of the RsmA/CsrA family mediate the post-transcriptional regulation of diverse genes required for bacterial metabolism and virulence. These include genes for induction of carbon starvation response (Romeo et al., 1993, Sonnleitner et al., 2011, Yang et al., 1996), motility (Ang et al., 2001, Liaw et al., 2003, Wei et al., 2001), quorum sensing (cell-to-cell communication) (Chatterjee et al., 1995, Cui et al., 1995), biofilm development (Wong and Akerley, 2005), and the production of secondary metabolites and virulence factors (Barnard et al., 2004, Blumer et al., 1999, Heurlier et al., 2004, Pessi et al., 2001). In the Gram-negative pathogen, *Pseudomonas aeruginosa*, RsmA modulates the expression of ∼9% of the 5,570 predicted open reading frames in the PAO1 genome (Brencic and Lory, 2009, Burrowes et al., 2005). This occurs either directly (by binding their mRNAs and affecting translation rates and stabilities) or indirectly (by regulating the intracellular and intercellular signaling circuitry that modulates the expression of further subsets of genes).

RsmA homologs can negatively regulate translation by binding to multiple sites of the 5′-untranslated region (5′-UTR) close to the Shine-Dalgarno sequence and prevent ribosomal binding (Baker et al., 2002, Dubey et al., 2005). RsmA/CsrA proteins usually act as translational repressors but can also function as activators via processes, which involve binding to the untranslated mRNA leader, with consequent effects on RNA turnover and/or translation (Romeo et al., 2013). RsmA activity is regulated by two small, regulatory RNAs (RsmY and RsmZ) that sequester multiple copies of the protein preventing binding to lower-affinity mRNA targets (Burrowes et al., 2005, Heeb et al., 2002, Heurlier et al., 2004, Liu and Romeo, 1997, Liu et al., 1998, Ma et al., 2001, Valverde et al., 2003, Weilbacher et al., 2003). Thus, the effects on the Rsm regulon depend on the relative levels of these noncoding sRNAs, which facilitate rapid responses to changes in the local environment.

Structures of RsmA orthologs (CsrA, RsmA, and RsmE) reveal a homodimer consisting of a domain-swapped five-stranded β sheet with α helices protruding from the C-termini (Gutiérrez et al., 2005, Heeb et al., 2006, Rife et al., 2005, Schubert et al., 2007). In vitro selection experiments have suggested that the protein dimer binds preferentially to a 5′-RUACARGGAUGU RNA loop motif (Dubey et al., 2005). Subsequent NMR studies of RsmE from *Pseudomonas protegens* CHA0 (previously *P. fluorescens* CHA0), which regulates biocontrol factors, have revealed molecular details of the complex with an RNA hairpin target from the *hcnA* gene encoding hydrogen cyanide synthase subunit A (Schubert et al., 2007). The structure of the complex and subsequent modeling studies suggest how an RsmE homodimer with two identical binding sites interacts with tandem GGA-containing RNA motifs, with avidity effects enhancing the binding affinity for the unstructured ribosome binding site in the 5′-UTR (Dubey et al., 2005, Lapouge et al., 2013, Mercante et al., 2009).

The Rsm/Csr systems described in different bacteria contain varying numbers of RsmA orthologs ranging from one to three nonidentical copies. Variations in sequence, structure, RNA-binding affinities, and specificities within a single cell appear to facilitate tight gene-specific control at the global post-transcriptional level; however, the molecular basis of this remains largely unexplored. Of three RsmA homologs (A1, A2, and A3) in *Pseudomonas entomophila*, only the first two appear to be functionally equivalent (Vallet-Gely et al., 2010). *P. protegens* CHA0 expresses two protein homologs (RsmA and RsmE) and three sRNAs (RsmX, RsmY, and RsmZ), with the protein homologs differentially expressed (Kay et al., 2005, Reimmann et al., 2005). In *P. aeruginosa*, the sRNA RsmY, but not RsmZ, can be bound and stabilized by the RNA chaperone protein Hfq to more effectively block the action of RsmA (Sorger-Domenigg et al., 2007).

The RsmA/Y/Z system of *P. aeruginosa* strain PAO1 is the most intensively investigated among the pseudomonads, but the annotated genome identifies only one RsmA homolog. However, through phenotypic complementation of a *P. aeruginosa* PAO1 *rsmA* mutant, we discovered a protein, which we termed RsmN. This protein has a unique sequence compared with all other CsrA/RsmA orthologs identified to date. Here we demonstrate that RsmA and RsmN have functionally overlapping roles in the post-transcriptional control of bacterial behavior. We have solved the crystal structure of RsmN at 2.0 Å resolution and report a distinctly different polypeptide fold to the domain-swapped dimeric structure described for RsmA homologs (Gutiérrez et al., 2005, Heeb et al., 2006, Schubert et al., 2007). Despite the unique fold, the overall β sheet topology of the dimer required for RNA recognition is highly conserved. We show that the RsmN dimer binds to 5′-ANGGA consensus RNA motifs in both structured hairpin loops and in flexible disordered mRNAs present in regulatory sequences overlapping the ribosome binding site and in the noncoding sRNAs (RsmY and RsmZ). To elucidate the molecular details of the interaction, we solved the structure at 3.2 Å resolution of the RsmN dimer complex with two 16-mer RsmZ regulatory sRNA hairpins. The structure reveals how sequence differences between RsmN and RsmE/A that result in topological changes in the dimer structures translate to compensatory differences in sequence-specific RNA recognition.

## Results

### Discovery of RsmN, a Member of the Rsm/CsrA Family of RNA-Binding Proteins

**Figure 1:**
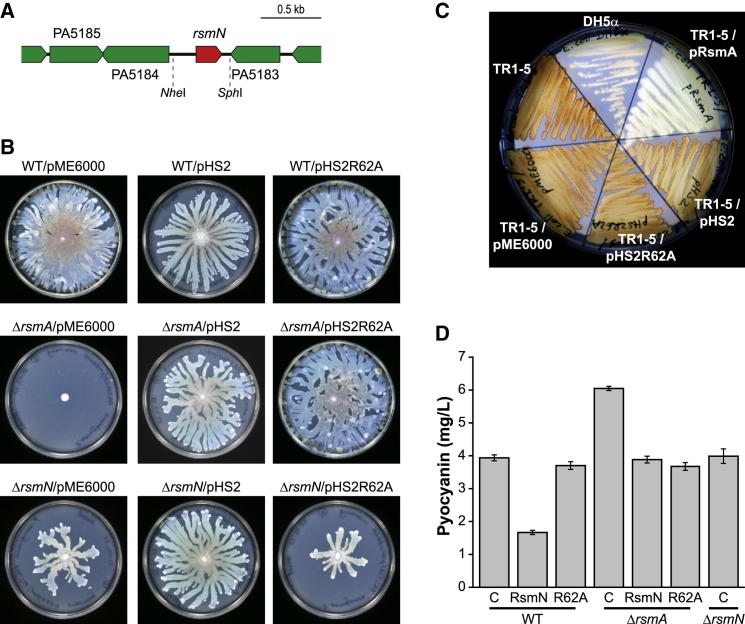
Genetic Context and Phenotypes Associated with *rsmN* (A) Schematic representation of the *P. aeruginosa* PAO1 genomic bank DNA fragment that restores the swarming phenotype of a PAO1 *rsmA* mutant. The intergenic region (red) between the genes PA5183–5184 encodes the novel RsmA/CsrA homolog RsmN in the antisense direction. (B) Swarming motility assay of the *P. aeruginosa* PAO1 wild-type (top), *rsmA* mutant (middle), and *rsmN* mutant (bottom), respectively, complemented with the vector pME6000, the same vector expressing *rsmN* (pHS2), or the *rsmN* R62A mutant. Both RsmN and the RsmN R62A variant can fully restore swarming in the *rsmA* mutant. Plasmid-borne *rsmN*, but not the *rsmN* R62A mutant gene, complements the reduced swarming phenotype of an *rsmN* mutant. (C) Repression of glycogen synthesis in the *E. coli csrA* mutant TR1-5 by *P. aeruginosa* RsmA but not by RsmN or the RsmN R62A mutant. *E. coli* TR1-5 overproduces glycogen on Kornberg medium as detectable by iodine staining. Glycogen production was assayed for strain TR1-5-carrying plasmids pRsmA, pHS2, pHS2R62A, or the empty expression vector pME6000. (D) Pyocyanin production was assayed for the wild-type strain PAO1 (WT), the *rsmA* mutant carrying the empty vector pME6000, pHS2, or pHS2R62A and the *rsmN* mutant. Each value is the average from three different cultures ± SD.

**Figure 2:**
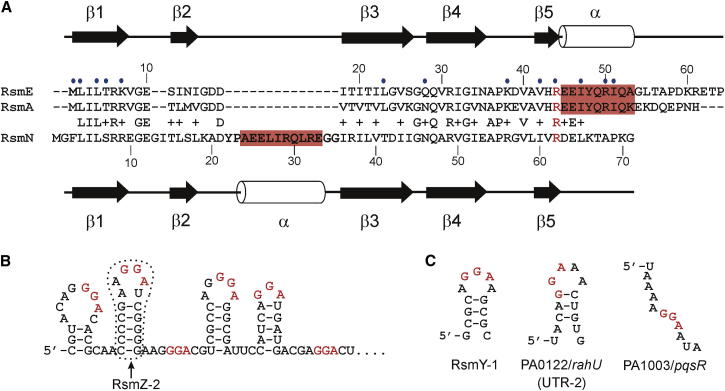
Secondary Structures of RsmA, RsmN, and the RNAs Assessed for Binding (A) Sequence alignment for RsmE, RsmA, and RsmN orthologs showing sequence similarities, secondary structure alignment, insertions/deletions in the RsmN sequence, and residues in the RsmE structure involved in RNA binding contacts (blue circles). (B) A portion of the sequence of the sRNA *rsmZ* showing potential GGA binding sites (red) and, in particular, the hairpin loop motif RsmZ-2 used in structural studies. (C) Hairpin loop structures formed by the regulatory sRNA RsmY-1, and by UTR-2 of PA0122/*rahU*, and the unstructured purine-rich PA1003/*pqsR* sequence.

The annotated genome of *P. aeruginosa* strain PAO1 (Stover et al., 2000) shows only a single RsmA protein. In our *P. aeruginosa* PAO1 laboratory subline, mutation of *rsmA* results in the complete loss of swarming motility (Heeb et al., 2006, Heurlier et al., 2004). In an attempt to discover additional components within the RsmA regulatory cascade, we transformed a random plasmid library containing 2-4 kbp PAO1 chromosomal DNA fragments into the *rsmA* mutant and screened the colonies obtained for restoration of swarming. One of the clones identified as capable of partially restoring motility carried plasmid pPAMMB-16 (Table 1), which was found to contain a chromosomal DNA insert incorporating genes PA5181 to PA5186. An in silico analysis undertaken to predict the gene(s) present on the genomic insert most likely to contribute to the RsmA regulatory pathway revealed that the 513 bp intergenic region between open reading framesPA5183 and PA5184 contained an unannotated open reading frame encoding a 7.8 kDa protein (Figure 1A). We termed this gene *rsmN* due to its “novel” sequence and to highlight its more distant relatedness to the *rsmA*/*rsmE* homologs (Figure 2A).

**Table 1:** Strains, Plasmids, and DNA Oligonucleotides Used in This Study

Strain/Plasmid/Primers	Relevant Characteristics	Source/Reference
***E. coli***		
DH5α	laboratory strain for cloning and plasmid maintenance	Stratagene
S17-1λ*pir*	conjugative strain for the mobilization of suicide plasmids	(Simon et al., 1983)
C41(DE3)	OverExpression C41(DE3), T7 gene expression strain	Lucigen

***P. aeruginosa***		

PAO1	wild-type, parent of the strains below	Laboratory collection
PAZH13	Δ*rsmA*, in-frame deletion mutant	(Pessi et al., 2001)
PALT16	*ΔrsmN*, derived from PAO1 and pMM33	This study

**Plasmids**		

pME6000	pBBR1MCS derivative cloning vector (Tc^R^)	(Maurhofer et al., 1998)
pPAMMB-16	pME6000 with a 2.5-kb PAO1 chromosomal DNA insert spanning from PA5182 (partial) to PA5186 (partial)	This study
pHS2	derivative of pPAMMB-16 with insert reduced to the *Nhe*I-*Sph*I 0.47-kb fragment carrying *rsmN*	This study
pHS2R62A	equivalent to pHS2 but carrying a CGC to GCC (R62A) substitution in *rsmN*	This study
pMM33	pDM4 derivative for the in-frame deletion of *rsmN* by allelic exchange, constructed by joining PCR products obtained with primers RSMNU and RSMND	This study

**PCR Primers (5′**–**3′)**		

RSMNUF	TATCTCGAGTACTGGACCAGCTTGTTCG, upstream forward primer for the in-frame deletion of *rsmN*, *Xho*I site	
RSMNUR	TATGAATTCACCCATGTTCCGCGTCCTT, upstream reverse primer, counterpart of RSMNUF, EcoRI site	
RSMNDF	TATGAATTCGGCTGACGAACGGTAGAAA, downstream forward primer for the in-frame deletion of *rsmN*, EcoRI site	
RSMNDR	TATTCTAGATGTGCGAACGACCGTATTTC, downstream reverse primer, counterpart of RSMNDF, *Xba*I site

RsmN is slightly longer than most RsmA/CsrA proteins (71 versus 61 amino acid residues; Figure 2A). The striking observation is that RsmN lacks the C-terminal residues that form the α helical structure in RsmA. Instead, where RsmA has a short linker between β strands 2 and 3, RsmN has a 16-residue loop insertion with a high helical propensity. Allowing for the inserted loop residues, the RsmN and RsmA sequence identity is 34%, with 52% similarity. BLAST searches revealed that the *rsmN* gene is highly conserved and virtually identical (97%) in each of the 11 completed *P. aeruginosa* strain genomes currently available. In strains PA14 and LESB58, RsmN is identical to that of PAO1, whereas in PA7, RsmN is essentially identical in sequence but is 73 rather than 71 amino acids in length and diverges at the seven C-terminal residues.

### Impact of *rsmN* on Swarming Motility, Glycogen Accumulation, and Pyocyanin Production

To determine whether *rsmN* was responsible for the ability of pPAMMB-16 to restore swarming, *rsmN* was cloned into pME6000 to generate pHS2 and introduced into the *P. aeruginosa rsmA* mutant. Figure 1B shows that swarming motility was restored by providing in *trans* multiple copies of *rsmN* in the *rsmA* mutant. Furthermore, deletion of *rsmN* in an otherwise wild-type background reduced, but did not abolish, swarming motility (Figure 1B). These data suggest that RsmN can compensate for the loss of RsmA and that both RsmA and RsmN proteins are involved in the control of swarming. Furthermore, in RsmA we have shown that residue Arg44 is essential for RNA binding and functionality in the swarming assay (Heeb et al., 2006). To determine whether the equivalent residue (Arg62) in the larger RsmN protein is also essential, we constructed a plasmid expressing an R62A variant of RsmN. When the mutated *rsmN* gene was introduced into the *P. aeruginosa rsmA* mutant, swarming motility was fully restored, indicating that the equivalent arginine residue is not essential for RsmN functionality in this assay. However, while the reduced swarming of an *rsmN* mutant can be restored by the provision of a plasmid-borne *rsmN* gene, under the same conditions the RsmN R62A construct is unable to restore this phenotype (Figure 1B). These data suggest that RsmN and RsmA have distinct but overlapping contributions to swarming motility.

RsmA/CsrA orthologs characteristically complement the *csrA* mutation in *Escherichia coli* strain TR1-5 responsible for the overproduction of glycogen (Heeb et al., 2006, Romeo et al., 1993). Figure 1C shows that, in contrast to RsmA, neither RsmN nor the RsmN R62A mutant could repress glycogen biosynthesis in TR1-5.

Mutation of *rsmA* in *P. aeruginosa* results in the enhanced production of the blue-green phenazine pigment, pyocyanin (Heeb et al., 2006). A plasmid-borne copy of *rsmN* reduced pyocyanin back to wild-type levels in the *rsmA* mutant but also reduced production by the wild-type strain (Figure 1D). However, although introduction of the *rsmN*R62A mutant gene into the *rsmA* mutant also reduced pyocyanin levels, it did not impact on pyocyanin production by the parent strain. Mutation of *rsmN* had no effect on pyocyanin production (Figure 1D).

### Crystal Structure of the RsmN Dimer

**Figure 3:**
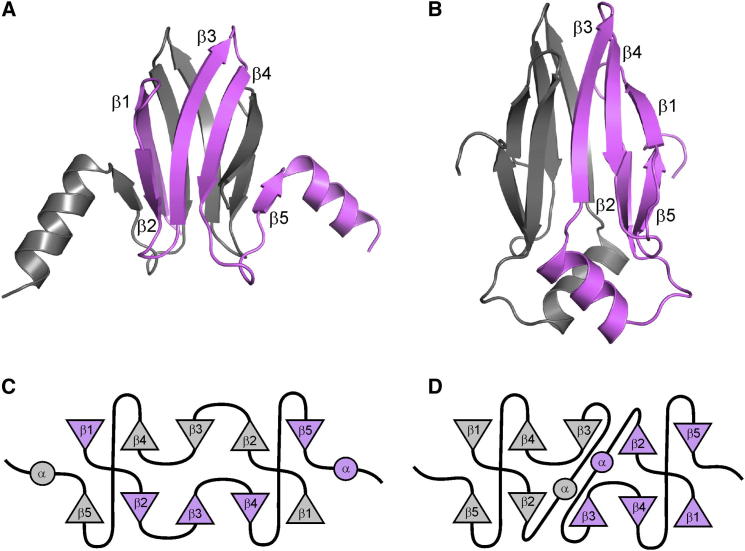
Structural Comparison of RsmN and RsmA (A and B) Cartoon diagrams of the crystal structures of *P. aeruginosa* PAO1 RsmA (A) and RsmN (B) showing the differences in the polypeptide chain topology (different monomers colored gray and pink) and the relative positions of the two α helices. Strands in one of the monomer units of each structure are labeled. The RsmN dimer is stabilized by mutual contacts between the two α helices, which in turn pack against the β sheet to expand the hydrophobic core (see also Figure S2). (C and D) The helices of RsmA make few stabilizing contacts to the dimer. The topology maps of RsmA (C) and RsmN (D) show domain swapping of β strands in the RsmA dimer but docking of the two folded monomeric units for RsmN. See also Figures S1 and S2.

To gain further insights into the structure and function of RsmN, we expressed and purified from *E. coli* a His-tagged protein construct and confirmed by gel filtration analysis and ESI-MS that RsmN forms a dimer (Figure S1 available online). The crystal structure of RsmN was solved to 2.0 Å resolution (Figure 3) by molecular replacement using the *P. aeruginosa* RsmA crystal structure (PDB code 1VPZ). The asymmetric unit contains a single RsmN molecule, with the electron density allowing model building of the first 66 residues from RsmN out of a total of 71 (Table 2). As with RsmA, the C terminus of the RsmN polypeptide chain is unstructured and highly flexible. The RsmN structure is distinct and completely novel compared with those of all other RsmA/CsrA orthologs and reveals that the 16-residue insert between β strands 2 and 3 has the effect of redirecting the polypeptide chain to give a unique dimer structure.

**Table 2:** Data Collection and Refinement Statistics

	RsmN-RsmZ-2 Complex	RsmN
Space group	P43 2 2	P31 1 2
Unit-cell parameters (Å, °)	a = 83.7, b = 83.7, c = 94.2	a = 41.1, b = 41.1, c = 72.5
	α = 90, β = 90, γ = 90	α = 90, β = 90, γ = 120
Resolution limit (Å)	30.0–3.2 (3.4–3.2)	30.0–2.0 (2.1–2.0)
No. of observed/unique reflections	51,980/5,917	47,667/7,223
Completeness^a^ (%)	99.5 (97.7)	98.9 (92.7)
Data redundancy	8.8 (6.1)	6.6 (3.6)
R_merge_ (%)	8.5 (67.5)	6.9 (40.6)
Mean I/σI	18.6 (2.4)	13.5 (3.1)
R_work_/R_free_	0.220/0.320	0.218/0.314
Rms bond lengths (Å)	0.013	0.024
Rms bond angles (°)	1.718	2.429
No. of protein atoms	1,010	529
No. of RNA atoms	688	0
Residues in Ramachandran plot regions (%)		
Most favored	87.2	96.9
Additional allowed	12.8	3.1

a Values in parentheses represent the outer shell.

The RsmN dimer consists of a very similar ten-stranded interface held together by a sandwich of two five-stranded β sheets assembled in the same order: β5-β2-β3-β4-β1. The structure lacks the α helix at the C terminus, but instead, the 16-residue insert adopts a well-defined α helix between β strands 2 and 3 (Figure 3B), with the sequences represented as β1-β2-α-β3-β4-β5 and β1-β2-β3-β4-β5-α, respectively, for RsmN and RsmA. However, as evident in the topology maps (Figures 3C and 3D), these structural elements assemble differently in each protein dimer. Each five-stranded β sheet in the structure of the RsmA orthologs consists of three central strands from polypeptide chain A and two outer strands from the second chain B to give two five-stranded β sheets with the composition β5_A_-β2_B_-β3_B_-β4_B_-β1_A_ and β5_B_-β2_A_-β3_A_-β4_A_-β1_B_ with swapping of the N- and C-terminal strands (β1 and β5) completing the β sheet (Figures 3A and 3C). In this arrangement, the loops connecting β1-β2 and β4-β5 are key “hinges” in directing the polypeptide chain to cross between the two β sheets. In RsmN, the noticeable difference is that the insertion of the α helix between β2-β3 sterically prevents these consecutive β strands from lying adjacent and antiparallel to each other within the same sheet. Instead, the α helix forces the polypeptide chain to cross over to form part of the other β sheet, such that the RsmN β sheets are composed as follows: β5_A_-β2_A_-β3_B_-β4_B_-β1_B_ and β5_B_-β2_B_-β3_A_-β4_A_-β1_A_ (Figures 3B and 3D). Thus, the overall folding topology of the two polypeptide chains and their dimerization interface is altered by the presence of the α helical insert, making the dimer interface no longer domain swapped but the product of two docked folded monomers (Figure 3A versus Figure 3B).

### The RsmN α-Helix Contributes Residues to the Hydrophobic Core

The dimerization interface of RsmA spans the entire hydrophobic core buried between the two β sheets. However, the C-terminal α helices protrude from the structure and form relatively few contacts with the core β sheet (Figures 3A and 3C). NMR relaxation data show that the α helices are dynamic in the NMR structure of CsrA (Gutiérrez et al., 2005). In contrast, the β2-α-β3 loop in RsmN crosses the β sandwich, and the protein chain folds back on itself to form a more compact monomeric unit with a much reduced dimerization interface, consisting of the edges of β2, the α helix, and β3 (Figures 3B and 3D). Surface area analysis shows that RsmA buries at ∼4,453 Å^2^ in its interwined dimer structure; however, this is significantly smaller for the RsmN dimer (2,841 Å^2^). To compensate, the amphipathic helix of RsmN forms an integral part of the hydrophobic core of the dimer structure largely via residues Leu27, Ile28, and Leu31, which form mutually stabilizing helix helix interactions and contacts with residues at the ends of the β sheet and adjacent loops (Leu18, Ile36, Pro55, and Val58) (Figure S2).

### Sequence Alignment and Potential Structural Differences in RNA Recognition

The sequence alignment for RsmE (from *P. protegens*) with RsmA and RsmN from *P. aeruginosa* reveals high sequence identity or conservation among those residues, such as Arg44, which play a functional role in RNA binding (Heeb et al., 2006) as identified in the RsmE complex with an *hcnA* mRNA hairpin (Schubert et al., 2007) (Figure 2). Furthermore, we have shown experimentally that the RsmN residue Arg62 (equivalent to Arg44) is required for complementation of swarming motility in a *P. aeruginosa rsmN* deletion mutant (Figures 1 and 2). There are, however, several notable structural differences. In the RsmE homolog, residues within the α helix and at the C terminus were shown to be important in forming a hydrophobic patch (through Ile47, Ile51, Ala57, and Pro58), which accommodates the looped-out C9 base in the 6-nucleotide recognition motif (ACGGAN). Moreover, the side chain of Arg50 was implicated in binding the phosphate backbone between A8 and C9 to reinforce this interaction. However, RsmN lacks the C-terminal α helix found in the RsmA orthologs, and Arg50 is substituted at the equivalent position for Ala68 in the RsmN structure. Although the nine C-terminal amino acid residues of RsmA (Lys53 to His61) can be deleted without loss of activity, any further truncations into the α helix result in the complete loss of function in terms of both swarming and glycogen assays (Kuehne, 2008).

**Figure 4:**
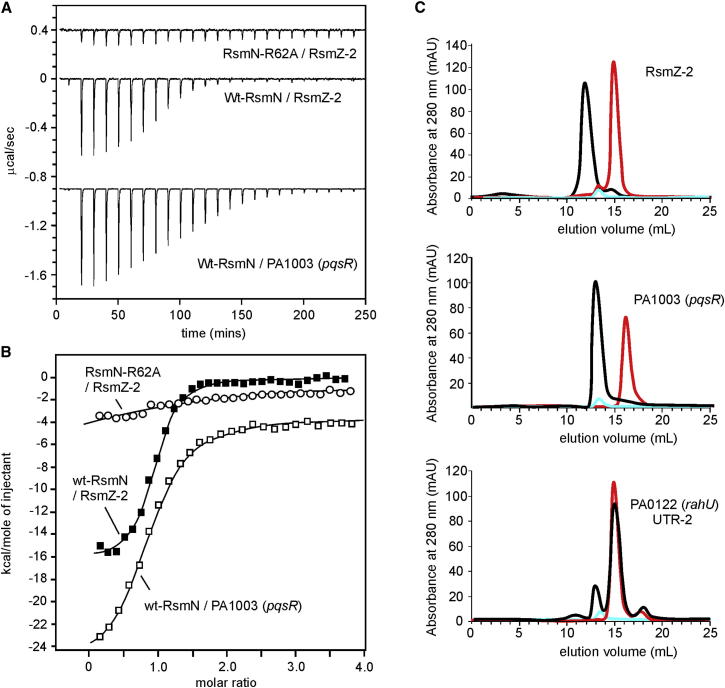
RsmN-RNA Binding Analysis (A) ITC profiles for binding of RsmN and the RsmN R62A mutant to RsmZ-2 and the ribosome binding site of PA1003/*pqsR*. (B) ITC binding isotherms constructed from the data in (A) and fitted to a 1:1 binding model. Measurements at 25°C in 50 mM NaCl, 25 mM potassium phosphate buffer, pH 7.0. The data in both plots are shifted apart on the *y* axis for clarity. (C) Binding interactions of RsmN determined qualitatively by analytical SEC showing a shift in retention time of the band for unbound RNA (red) to faster elution for the complex (black); protein alone is shown in blue. SEC profiles (from the top) are for RsmZ-2, the unstructured ribosome binding site of PA1003/*pqsR*, and the UTR-2 from the mRNA transcript of gene PA0122/*rahU*. The first two show complete conversion to the bound state; the latter shows a much weaker interaction and a small population in the bound state. Similar profiles are observed for the binding of RsmA (see Figure S4). See also Figures S3, S4, and S6.

Therefore, we first determined whether these structural differences have a significant impact on RNA-binding affinity. We chose a 16-nucleotide hairpin from the sRNA, RsmZ (Figure 2B), which antagonizes RsmA (Heurlier et al., 2004). RsmZ hairpin 2 (RsmZ-2) contains a native G-rich stem region with a predicted 5′-AAGGAU hexanucleotide loop that conforms to the consensus ANGGAN recognition motif. NMR studies confirmed that RsmZ-2 forms stable Watson-Crick hydrogen-bonded secondary structure (Figure S3). Analytical size exclusion chromatography (SEC) showed clear evidence for complex formation with RsmN. Quantitative analysis using ITC experiments recorded a *K*_d_ of 276 ± 25 nM (Figure 4). Comparisons with RsmA showed essentially identical affinity (*K*_d_ = 264 ± 43 nM) and SEC profiles (Figure S4) with both complexes described by a 1:1 binding stoichiometry, indicative of one hairpin binding to each half of the dimer.

### Structure of the RsmN-RsmZ Hairpin 2 Complex

**Figure 5:**
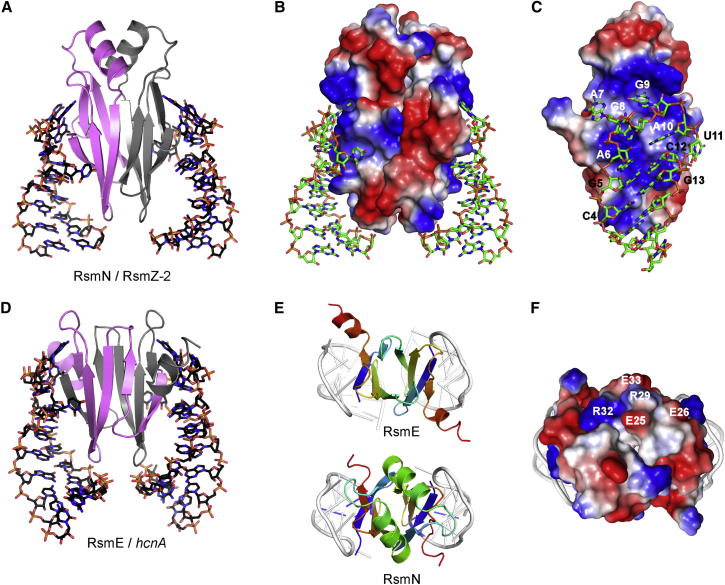
Structure of the RsmN Complex with the RsmZ-2 RNA Hairpin (A) Structure of the RsmN dimer complex with base specific contacts with the protein β sheet, showing the orientation of the two helices away from the binding interface. (B) Electrostatic surface representation showing RNA interactions with the positively charged faces of the dimer (blue, positive charge; red, negative charge). (C) Side on view of specific base contacts of one monomeric RsmN unit with the AAGGA recognition loop of RsmZ-2. (D) Corresponding structure to (A) of the RsmE domain-swapped dimer complex with *hcnA* showing contacts from both halves of the dimer to each RNA hairpin. (E) Top-down view of the two complexes showing the unique side-by-side arrangement of the two helices of the RsmN dimer in forming a putative binding surface (ribbon representation of the two dimers: blue, N-terminus; red, C-terminus). (F) Electrostatic potential surface of the RsmN dimer (same orientation as E) showing a charged surface patch formed by the side by side alignment of the two α helices (residues of one α helix are labeled); RNA hairpin shown in gray in both (E) and (F). See also Figure S5.

We determined the crystal structure of the RsmN/RsmZ-2 complex to 3.2 Å resolution (Figures 5A–5C). The asymmetric unit contains an RsmN dimer with residues 3-66 of the 71 residue construct observed in the electron density. RsmN does not have the domain-swapped dimer structure evident for the RsmA orthologs (Figure 3); and as a result, each RsmN monomer contacts just one of the bound RNA hairpin motifs through a highly basic surface comprising Arg9, Arg49, Arg56, and Arg62 (Figure 5C). In contrast, in the NMR structure of the RsmE/*hcnA* complex, each RsmE monomer is able to simultaneously interact with both RNA hairpins using the equivalent set of charged residues to RsmN to establish a similar set of RNA interactions (Figure 5D). The two α helices of the RsmN dimer do not contact the RNA but lie side by side to generate a unique distal polar surface involving charged residues Glu25, Glu26, Arg29, Arg32, and Glu33 orthogonal to the RNA binding surfaces (Figures 5E and 5F).

**Figure 6:**
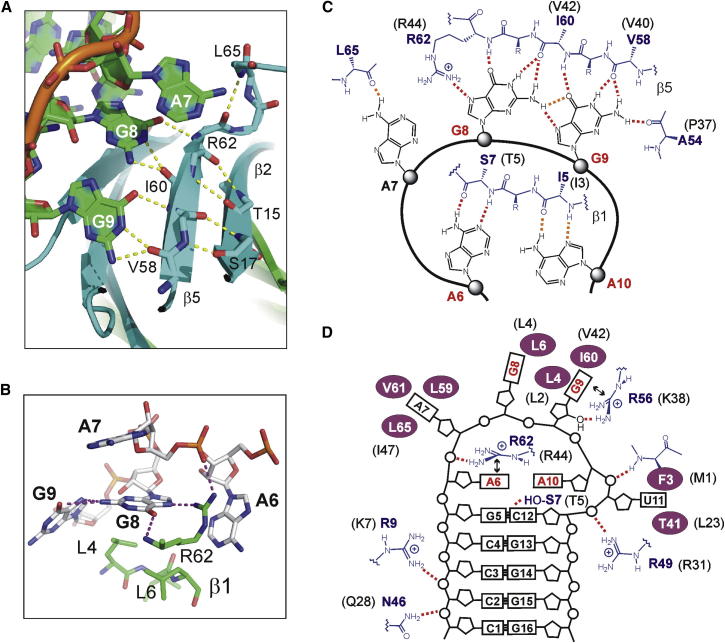
Key Features of Protein-RNA Recognition in the RsmN/RsmZ-2 Complex (A) Details from the X-ray structure showing base-specific multidentate hydrogen bonding interactions between G8 and G9 and backbone amide NH and CO groups of residues Val58 and Ile60 in strand β5, and the NH of Arg62 in the adjacent loop. (B) Side chain hydrophobic interactions of Leu4 and Leu6 in strand β1 with the faces of the purine bases of G8 and G9. Multiple interactions of the guanidinium side chain of Arg62 are evident with the phosphate backbone of A7 and the N7 of G8, which are enhanced by π-stacking of the guanidinium group against the face of A6. (C) Schematic representations of hydrogen bonding to the ANGGA recognition motif of RsmZ-2, showing mutually stabilizing hydrogen bonds between G8 and G9 that position the two guanines for intermolecular interactions through their Watson-Crick edges with residues in strand β5; A6 and A10 are recognized specifically through their Watson-Crick and Hoogsteen edges. (D) Other polar and hydrophobic side chain contacts from RsmN that stabilize the RsmZ-2 complex. The equivalent contact residues in the RsmE complex with *hcnA* are shown in (C) and (D) in parentheses and refer to contacts shown in Schubert et al. (2007) (their Figure 3E). See also Figure S5.

Sequence-specific recognition of the 5′-AAGGAU binding motif of RsmZ-2 is facilitated by positioning the consensus ANGGA motif within the flexible hairpin loop. Hydrogen bonding between the bases of G8 (N2) and G9 (O6 and N7) positions the “GG” motif in an orientation that presents the Watson-Crick edges of the two purines for a set of base-specific intermolecular hydrogen bonds through residues in strand β5 and the adjacent loop primarily through the amide groups in the polypeptide backbone (Figures 6A and 6C). Ile60 forms multidentate contacts with both G8 and G9, supported by other specific CO or NH interactions from Ala54, Val58, and Arg62 (Figure 6A). Stabilizing hydrophobic contacts are also evident on the protein surface with G8 buried against Leu6, and G9 against the side chains of Leu4 and Ile60 (Figure 6B). Overall, there is a high degree of similarity in the nature of the stabilizing contacts with those described in the RsmE complex with *hcnA* (Schubert et al., 2007).

Base-specific recognition is also evident for the two adenines within the consensus binding motif (A6 and A10 in RsmZ-2), which are positioned directly above the G5-C12 base pair at the end of the double-stranded stem region (Figure 5C). Again, specificity is mediated through amide backbone interactions with residues within strand β1. A6 is recognized by Ser7 through its Watson-Crick edge, whereas the interactions of A10 with Ile5 are mediated through the Hoogsteen edge of the purine base (Figure 6C). The numerous other electrostatic and hydrophobic side chain contacts that stabilize the complex are illustrated in Figures 6C and 6D. In particular, Arg62 in RsmN is a residue that is highly conserved across the family of RsmA orthologs and is shown to form multidentate stabilizing contacts in the RNA complex, including a face-to-face π-stacking interaction of the guanidinium group with A6 (Figure 6B). An RsmN R62A mutant, which fully retains the structural integrity of the dimer (Figure S5A), binds to RsmZ-2 with >1,000-fold lower affinity than *wt*-RsmN (Figure 4), consistent with the pivotal role of Arg62 in maintaining the RsmN phenotype (Figure 1B).

At the C terminus, where the RsmA orthologs have a short α helix, RsmN has a shallow hydrophobic patch involving Leu59, Val61, and Leu65, which binds A7 nonspecifically in an analogous fashion to the corresponding C9 of *hcnA* (Schubert et al., 2007). Substitution of Arg50 of RsmA/E with Ala68 at the equivalent position in RsmN would appear to delete a contact to the phosphate backbone; however, the ITC-derived binding affinities of RsmA and RsmN for RsmZ-2 appear to be unaffected, or compensated by other interactions.

### Context Dependence of the GGA-Binding Motif for RsmN

We investigated the importance of RNA secondary structure on RsmN binding by considering physiologically relevant sequences with an ANGGA motif identified via a sequence-based approach for prediction of CsrA/RsmA targets (Kulkarni et al., 2013). Their binding interactions with RsmN and RsmA were characterized using ITC and/or SEC methods (Figures 4 and S4). ITC analysis with the unstructured purine-rich 11-mer derived from the PA1003 (*pqsR*) gene transcript, which codes for the transcriptional regulator PqsR of the 2-alkyl-4-quinolone-dependent quorum sensing pathway (Heeb et al., 2011, Ilangovan et al., 2013), showed that RsmN bound with 3-fold lower affinity (K_d_ = 704 ± 59 nM) than to the RsmZ-2 hairpin (Figure 4). Further, NMR titration experiments of RsmN with both RsmZ-2 and PA1003 (*pqsR*) resulted in both complexes forming in intermediate to slow exchange with initial resonance broadening followed by sharpening at binding saturation. Moreover, qualitative NMR analysis of RsmN chemical shift perturbations in the formation of the two complexes suggests many similarities in the mode of interaction between the fully structured RsmZ-2 hairpin and the extended single-stranded PA1003 (*pqsR*) gene transcript (Figure S5B).

We also considered solvent accessibility of the GGA motif in the context of other hairpin loops. A hairpin from the regulatory sRNA, RsmY (RsmY-1), which forms a stable hairpin with a 5-nucleotide loop containing an exposed GGA motif (Figure 2C), binds to RsmN with an affinity of 526 ± 65 nM, 2-fold weaker than to RsmZ-2 (Figure S6). Although the structural details of this interaction remain to be determined, this particular motif suggests that RsmN has some tolerance to a smaller but flexible loop motif without significant loss of affinity. In contrast, a more compact hairpin sequence with a 4-nucleotide loop derived from the 5′-UTR of PA0122/*rahU* (UTR-2; Figure 2C) showed evidence of only weak binding to RsmN (or RsmA) by analytical SEC (Figures 4C and S4). In this case, the GGA motif is likely to be partially buried through base-pairing within the stem-loop, which precludes base-specific contacts of the type described in Figure 6.

## Discussion

The function of RsmA homologs from diverse bacteria is pivotal in facilitating rapid survival responses to hostile conditions and in regulating bacterial interactions with higher organisms (Heroven et al., 2012, Timmermans and Van Melderen, 2010). Pseudomonads carry in their genomes multiple nonidentical copies of the *rsmA* gene and, to add a further layer of complexity, have different functional properties within a given bacterial strain. Although the genome of *P. aeruginosa* was previously thought to contain only a single *rsmA* allele, through functional complementation of a *P. aeruginosa rsmA* mutant with respect to swarming motility, we discovered a novel RsmA ortholog homolog we termed RsmN. Although RsmN, like RsmA could restore swarming and repress pyocyanin production in a *P. aeruginosa rsmA* mutant, it is, unlike RsmA, unable to complement an *E. coli csrA* mutant with respect to glycogen accumulation. In contrast to *rsmA*, a deletion of *rsmN* reduces, but does not abolish, swarming and has no effect on pyocyanin production. Furthermore, site-specific mutation of Arg62 in RsmN and the equivalent Arg44 in RsmA, both of which are essential for RNA-binding, resulted in an RsmN R62A protein, which retained the ability to complement the swarming-negative and pyocyanin overproduction phenotypes of the *rsmA* mutant. RsmN R62A was, however, unable to fully restore the reduced swarming phenotype of a *P. aeruginosa rsmN* mutant. These data indicate that RsmA and RsmN are likely to have both overlapping and distinct post-transcriptional regulatory roles.

To gain further insights into RsmN functionality, we solved the crystal structure. Although the overall fold of the RsmN dimer is quite different from RsmA and does not form a domain-swapped structure, remarkably, the spatial relationship of many key residues within the dimeric structure is conserved for RNA hairpin recognition (Gutiérrez et al., 2005, Heeb et al., 2002, Rife et al., 2005, Schubert et al., 2007). A close network of polypeptide backbone amide groups, rather than residue side chains, provide sequence-specific contacts to the GGA recognition motif that are observed in both the RsmN and RsmE complexes (Figure 6). The fundamental difference is the repositioning of the single C-terminal α helix of RsmA within the internalized β2-β3 loop sequence, which has the effect of redirecting the polypeptide backbone to change the topology of the dimer. Whereas the C-terminal “winged-helix” model of RsmA shows that the helix plays little role in maintaining the overall structural integrity of the dimer, the role of the α helix in RsmN appears to be critical in stabilizing the hydrophobic core of the protein by extending the dimer interface.

The RNA hairpin RsmZ-2 forms five G-C base pairs imparting structural stability to the double-stranded stem region. However, the majority of specific contacts in the RsmN complex are associated with the AAGGAU loop sequence. Only one interaction of significance, from the side chain hydroxyl group of Ser7, is involved in specific recognition of the C12 amino group of the G5-C12 base pair in the capping position at the end of the double-stranded stem region. In the RsmE/*hcn*A complex, Thr5 at the same position makes similar contacts with the C7-G14 base pair. However, the preceding U6-A15 base pair in the RsmE/*hcn*A structure is also described as forming specific base interactions with Gln29 and Arg31 (Schubert et al., 2007). Both of these residues are conserved in RsmN (Gln47 and Arg49), but neither forms close contacts with the C4-G13 base pair, although unresolved solvent-mediated interactions are a possibility. Thus, relatively high-affinity binding would appear to be possible in short RNA sequences, which are not necessarily stabilized by extended hairpin stem regions. Our analysis of the ANGGA recognition motif in short unstructured RNA sequences incorporating the ribosome binding site for PA1003 (*pqsR*) shows that both RsmN and RsmA bind effectively with a modest 2- to 3-fold lower affinity than to the highly structured hairpin RsmZ-2 (Figure 4).

The functional significance of the major reorganization of the RsmN fold to accommodate the central α helical motif into the dimer structure, while conserving the mode of RNA interaction, is intriguing. The presentation of the α helix on a distinct and sterically unhindered face of the RsmN dimer generates a potentially new interaction surface with the two helices uniquely aligned side by side (Figures 5E and 5F). The combination of acidic and hydrophobic residues (EEIYQRIQ in RsmA/E and EELIRQLRE for RsmN) is similar in the two α helical segments. In the RsmN dimer, these residues are brought together close to the dimer axis presenting a highly charged patch on a unique surface not present in RsmA.

**Figure 7:**
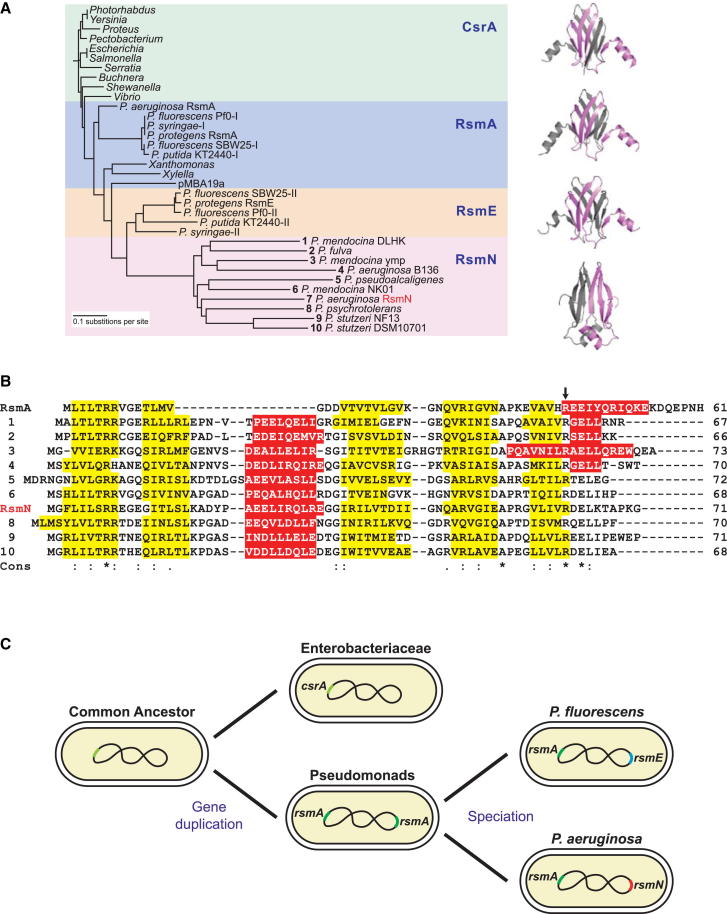
Phylogenetic Analysis of the Evolution of RsmN from RsmA Orthologs (A) Phylogenetic dendrogram of CsrA, RsmA, RsmE, and RsmN orthologs and their corresponding crystal structures. The dendrogram (FigTree) was derived from a ClustalW sequence alignment of CsrA homologs found in *Enterobacteriaceae* (green background), RsmA from various γ-proteobacteria closely related to *P. aeruginosa* (blue background), RsmE from *P. protegens* and related species (orange background), as well as the available orthologs of *P. aeruginosa* RsmN (pink background, numbered one through ten). Accession numbers of the sequences used are indicated in Table S1. (B) ClustalW alignment of the amino acid sequences of the 10 RsmN orthologs and RsmA from *P. aeruginosa*. Secondary structure predictions (YASPIN) of α helices and β-strands are highlighted in red and yellow respectively. Cons: amino acids conservation within the RsmN sequences, where ^∗^, complete; :, high; ., moderate conservation, respectively. The conserved Arg62 (Arg44 in RsmA) is indicated by an arrow. (C) Probable phylogeny of RsmN. From a common ancestor, gene duplication created a branch distinguishing the *Enterobacteriaceae*, which carry a single chromosomal homolog of *csrA*, from the Pseudomonads, which encode at least two alleles. Following speciation, one allele was conserved as RsmA while duplicated orthologs diverged independently toward *rsmE* or *rsmN* in the *P. fluorescens* and *P. aeruginosa* phylogenetic groups.

Given the similarity of the RsmA and RsmN sequences, the question arises as to the origin and evolution of these two related RNA-binding proteins with different folds. Although RsmN appears to be highly conserved only within *P. aeruginosa*, searches in other available genomes identified that homologs of RsmN are widespread within the genus *Pseudomonas*. The sequences of the nine RsmN-like proteins found were aligned with the sequences of representative CsrA/RsmA/RsmE orthologs of various species, and this alignment was used to build a phylogram, which shows a distinct clustering of this group (Figure 7A; Table S1). An amino acid alignment of the sequences of these nine proteins with the sequences of *P. aeruginosa* RsmA and RsmN is shown in Figure 7B. In common with RsmN, these nine sequences have the ∼16 amino acid insert between β strands 2 and 3, and secondary structure predictions suggest that this will also form an α helix (red in Figure 7B). Each of the nine RsmN-like proteins has a conserved Arg that aligns with the RsmN Arg62, which is critical for RNA binding. Furthermore, all are truncated at the C terminus compared with RsmA. Additionally, the alignment in Figure 7B shows that another conserved feature in RsmN and RsmA are residues Arg8 and Glu64 (RsmN numbering). These two residues form an intrasubunit salt bridge in RsmN, but in the RsmA domain-swapped dimer this salt bridge is formed as an intersubunit dimer contact. A feature present in all ten RsmN sequences, but not in RsmA, is the Gly-Ile motif, which occurs at the C terminus of the inserted α helix, and this may facilitate the topological change in redirecting the polypeptide chain to form a unique protein fold. Future studies will explore whether the putative new binding surface on the inserted α helical face of the RsmN dimer contributes to a specialized gene regulatory function in *P. aeruginosa*. Finally, it is intriguing that *Pseudomonas* species appear to maintain RsmA with either RsmE or RsmN, such that the latter two orthologs probably arose and diverged following an *rsmA* gene duplication event (Figure 7C).

## Experimental Procedures

### Bacterial Growth and Maintenance

The strains and plasmids used in this study are listed in Table 1. Bacterial growth conditions are described in detail in the Supplemental Information.

### *P. aeruginosa* DNA Manipulation

To screen for genes capable of restoring swarming in a *P. aeruginosa rsmA* mutant, genomic DNA was prepared from *P. aeruginosa* as described previously (Gamper et al., 1992), partially digested by *Sau*3AI and 2-4 kb fragments cloned into pME6000. The resulting plasmids were transformed into the *P. aeruginosa rsmA* mutant strain PAZH13 and each clone screened for restoration of swarming, as described in detail in the Supplemental Information.

### Swarming, Glycogen Accumulation, and Pyocyanin Production Assays

Swarming motility and pyocyanin production by *P. aeruginosa* strains were examined as described previously (Heeb et al., 2006, Heurlier et al., 2004). The ability of RsmN and the RsmN R62A mutant to complement the *csrA* mutation and repress glycogen production in *E. coli* strain TR1-5 was assayed by iodine staining as described previously (Romeo et al., 1993).

### Protein Production and RNA Preparation

The pET-28b(+) expression system (Novagen) was used to express His-tagged RsmA (His_6_-Thb-RsmA) and RsmN (His_6_-Thb-RsmN proteins) within host *E. coli* C41(DE3) cells, where “Thb” indicates an LVPRGS thrombin recognition sequence. Protein purification protocols, characterization, and isotopic labeling methods for NMR studies are described in the Supplemental Information.

Short RNA oligonucleotides were purchased from Dharmacon (Thermo Scientific), deprotected according to the manufacturer’s instructions, lyophilized, and stored at −20°C. RNA stock solutions (∼1 mM) were prepared from the lyophilized stocks. RNA hairpin formation was induced by thermally unfolding the RNA molecules at 95°C for 1 min, with subsequent cooling and reannealing monitored by NMR.

### Biophysical Characterization of Protein Structure and RNA Interactions

A combination of analytical SEC, ITC, and NMR spectroscopy was used to confirm the multimeric state of RsmA and RsmN after purification and to characterize protein-RNA interactions. Details of the instrumentation used, experimental protocols, and sample preparation are described in detail in Supplemental Information.

### Crystallization, Data Collection, and Refinement

A detailed description of the experimental conditions for crystal growth, data collection, and refinement methods for RsmN and the complex with the RsmZ-2 hairpin motif are presented in the Supplemental Information. Crystallographic data analysis and refinement statistics are presented in Table 2.

### Validation, Display, and Deposition

Analysis of the stereochemical quality of the model was accomplished using the AutoDepInputTool (http://deposit.pdb.org/adit/), MolProbity (Chen et al., 2010). Protein quaternary structure analysis used the PQS server (http://www.ebi.ac.uk/pdbe/pqs/) (Henrick and Thornton, 1998). Structures were displayed using PYMOL (http://pymol.org).

## Accession Numbers

The coordinates and structure factors for RsmN and the RsmN dimer complex with the RsmZ-2 RNA hairpin have been deposited in the Protein Data Bank with ID codes 4KRW (RCSB ID code: rcsb079717) and 4KJI (RCSB ID code: rcsb079416), respectively.

## Supplemental Information

Document S1. Supplemental Experimental Procedures, Figures S1–S6, and Table S1
