# Supplementary material for: Structural Rearrangement in an RsmA/CsrA Ortholog of Pseudomonas aeruginosa Creates a Dimeric RNA-Binding Protein, RsmN
Source: Structure. 2013 Sep 3;21(9):1659–71. doi: 10.1016/j.str.2013.07.007 (PMC3791407; doi:10.1016/j.str.2013.07.007)
Supplement: Document S1. Supplemental Experimental Procedures, Figures S1–S6, and Table S1 [file mmc1.pdf]

Structure, Volume *21*

Supplemental Information

Structural Rearrangement in an RsmA/CsrA Ortholog of *Pseudomonas aeruginosa* Creates a Dimeric RNA-Binding Protein, RsmN

Elizabeth R. Morris, Gareth Hall, Chan Li, Stephan Heeb, Rahul V. Kulkarni, Laura Lovelock, Hazel Silistre, Marco Messina, Miguel Cámara, Jonas Emsley, Paul Williams, and Mark S. Searle

Inventory of Supplemental Information

Supplementary Figure 1 (related to Fig. 3):
ESI-MS of RsmN demonstrating formation of a stable dimer.

Supplementary Figure 2 (related to Fig. 3):
Structural analysis of the RsmN dimer interface showing fit to the electron density in the RsmN dimer structure, residue side chain packing and overlay of RsmN and RsmE structures.

Supplementary Figure 3 (related to Fig. 4):
NMR analysis of the RNA hairpin from RsmZ-2.

Supplementary Figure 4 (related to Fig. 4):
ITC and analytical size exclusion chromatography (SEC) analysis of binding of the RsmA dimer to various 5'-ANGGAN target RNA sequences.

Supplementary Figure 5 (related to Figs. 5 and 6):
^{1}H-^{15}N TROSY spectra of RsmN, R62A mutant and RNA bound complexes.

Supplementary Figure 6 (related to Fig. 4):
ITC and NMR analysis of RsmY-1 and its RsmN complex.

Supplementary Table 1 (related to Fig. 7):
Source of the CsrA, RsmA, RsmE and RsmN homologues used for the phylogenetic analysis.

Supplemental Experimental Procedures:
Detailed description of all techniques and methods used in this study.

Supplemental references:
References relevant to the Supplementary Experimental Procedures.

Supplemental Information

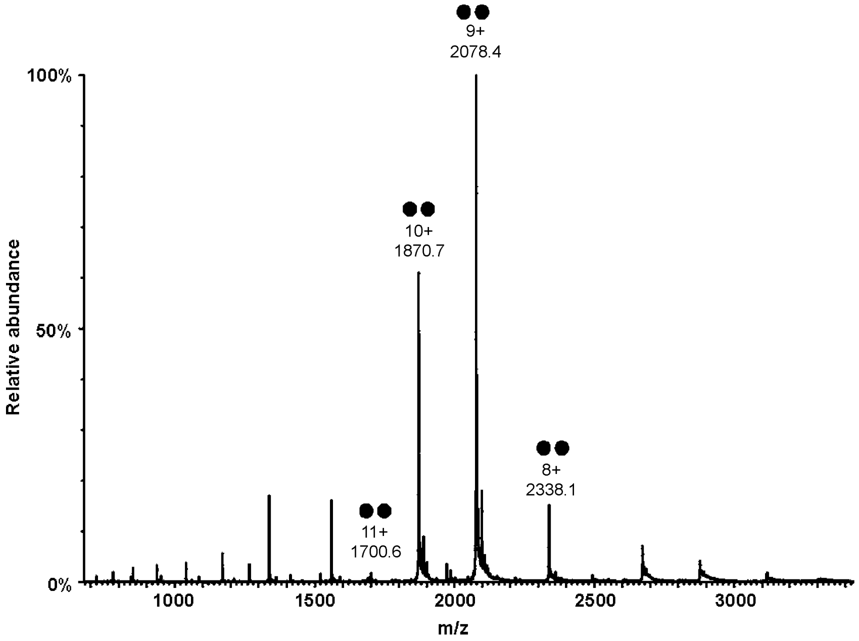

Supplementary Figure 1: ESI-MS analysis of the RsmN dimer

Electrospray ionisation mass spectrum (ESI-MS) showing the presence of predominantly the dimeric species (●●) in solution. Protein samples for ESI-MS were prepared by vigorous desalting through successive rounds of spin concentration using Vivaspin 500 (2 mL) centrifugal concentrators with a molecular weight cut-off (MWCO) of 3,000 Da (Sartorius Stedim). Lyophilised protein was first dissolved in 250 mM NH_4OAc and then exchanged twice into 25 mM NH_4OAc. ESI-MS measurements on native protein samples in 25 mM NH_4OAc were performed on a Waters SYNAPT High Definition Mass Spectrometer with a quadrupole time-of-flight mass analyser, which was calibrated using horse heart myoglobin (16,955 Da). Samples were infused into the electrospray source at 5 μL min-1 using a Harvard Apparatus syringe pump 22, model 55-2222, and a 100 μL Hamilton syringe. The capillary of the ESI source was held at 2.5-3 kV, with the instrument operating in positive-ion mode. A sample cone of 40 V and desolvation gas flow rate of 100 L h^{-1} were maintained throughout. The ESI mass spectra were recorded by scanning a mass/charge (m/z) range of 500-5,000. MassLynx (Waters) software was used to acquire and analyse data.

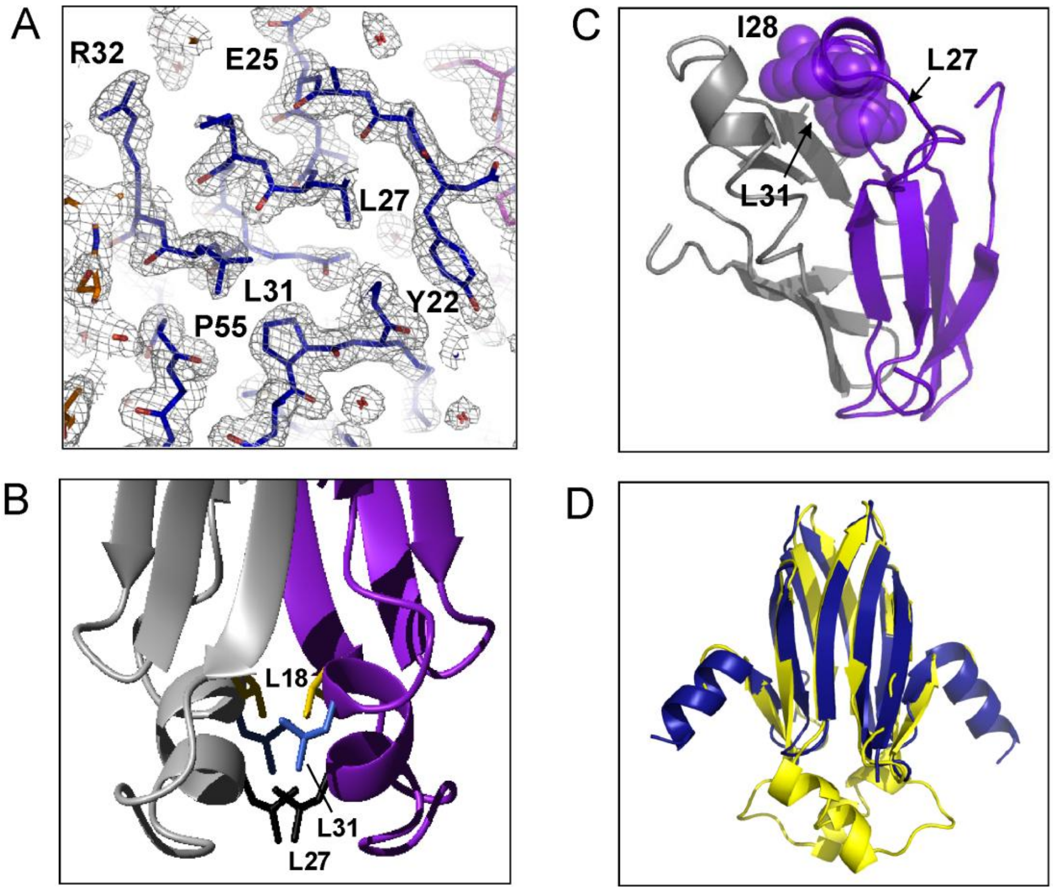

Supplementary Figure 2: Structural analysis of the RsmN dimer interface

(A) Portion of the electron density map for the RsmN dimer showing the fit of part of the helix that packs against the β-sheet to form part of the hydrophobic core..

(B) The amphipathic helices of RsmN form an integral part of the hydrophobic core of the dimer structure largely via residues Leu27, Ile28 and Leu31 which form mutually stabilising helix-helix interactions and contacts with residues at the ends of the β-sheet and adjacent loops (Leu18, Ile36, Pro55 and Val58; see **A**).

(C) Structure of the RsmN dimer showing the van der Waals surfaces of the helical residues Leu27, Ile28 and Leu31 in one half of the dimer which pack against equivalent residues in the other half.

(D) Overlayed structures of the RsmE and RsmN dimers showing conservation of the β-sheet structure, despite the different folds, but the completely different position and orientation of the helical motifs (RsmE, blue and RsmN, yellow).

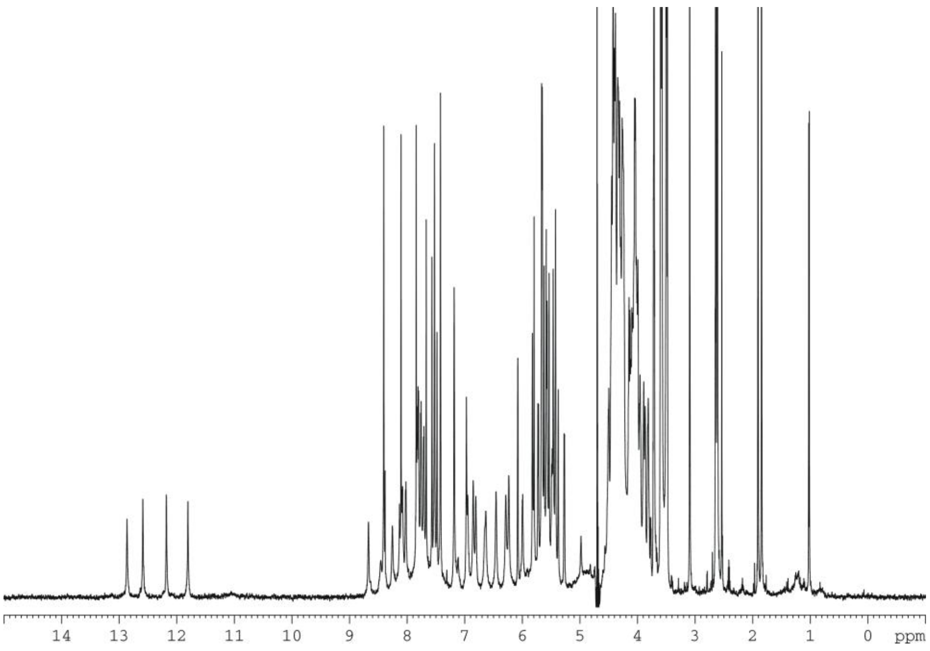

Supplementary Figure 3: NMR analysis of the RNA hairpin RsmZ-2

800 MHz ^{1}H NMR of RsmZ hairpin 2 (RsmZ-2) at 298K, phosphate buffered at pH 7.0, showing imino proton resonances in the 11.5 to 13 ppm region, consistent with hydrogen bonded Watson-Crick base pairing in a hairpin motif.

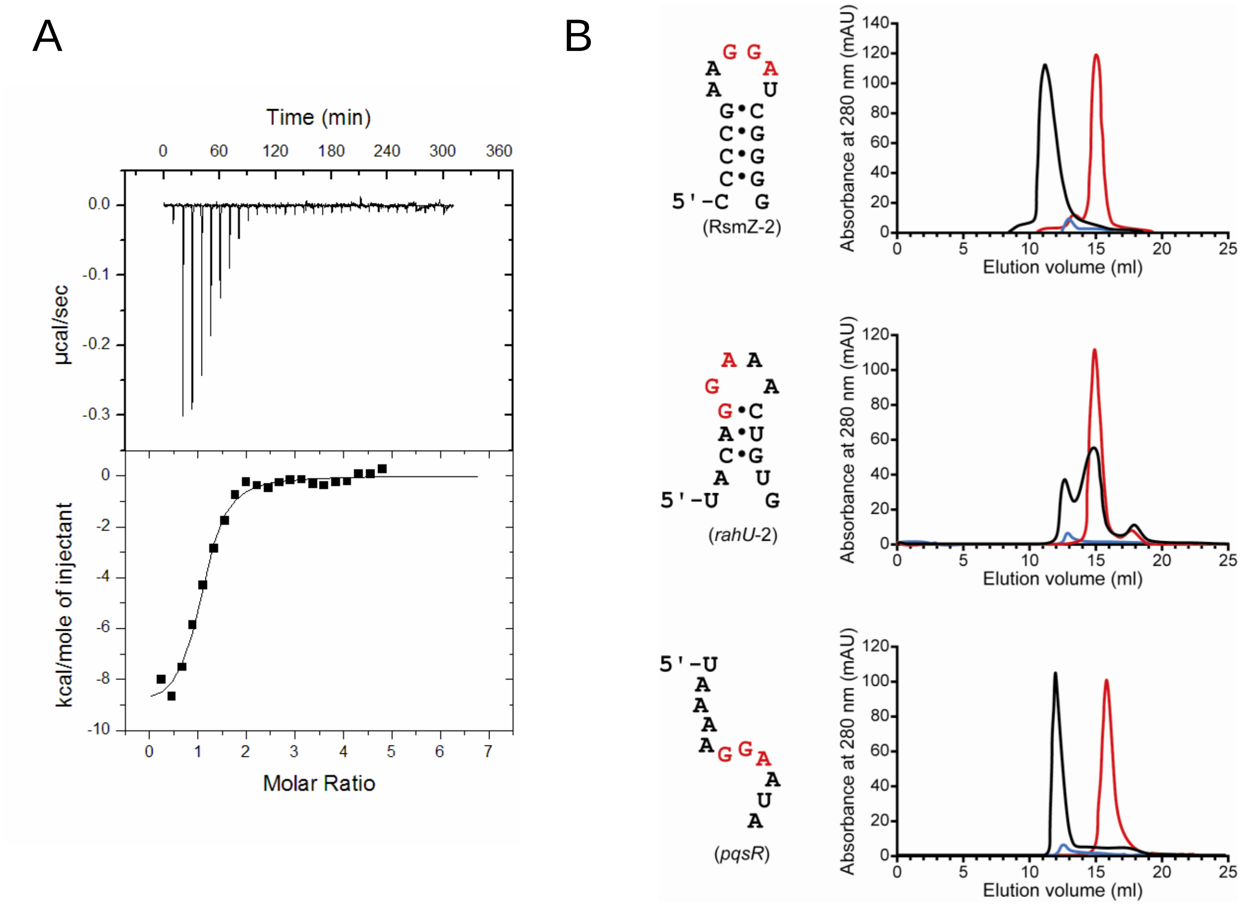

Supplementary Figure 4: ITC and analytical size exclusion chromatography (SEC) analysis of RNA hairpin binding to the RsmA dimer

(A) ITC analysis of the binding of RsmZ hairpin 2 to the RsmA dimer at 298K (K_d = 264 ± 43 nM). RNA (125 µM RNA, 25 mM potassium phosphate buffer pH 7, 50 mM NaCl) was titrated into a cell containing 1.424 mL protein (510 µM protein, 25 mM potassium phosphate buffer pH 7, 50 mM NaCl).

(B) RNA targets RsmZ-2 and UTR-2 (*rahU*-2) show evidence by NMR for folded hairpin structure, however, the purine-rich *pqsR* is unfolded. SEC data for RsmA showing strong binding interaction with RsmZ-2 and the unstructured purine-rich *pqsR*, but weak interaction with UTR-2 where only a small population of the bound state is visible at equilibrium under similar conditions (see experimental methods) (colour coding: red, RNA in isolation; blue, protein; black, complex formation).

A

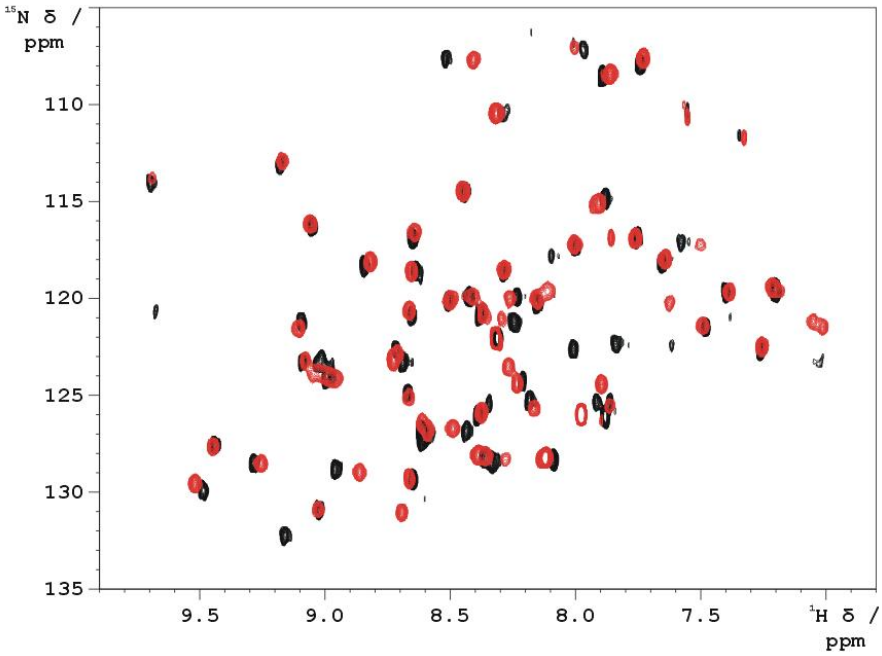

B

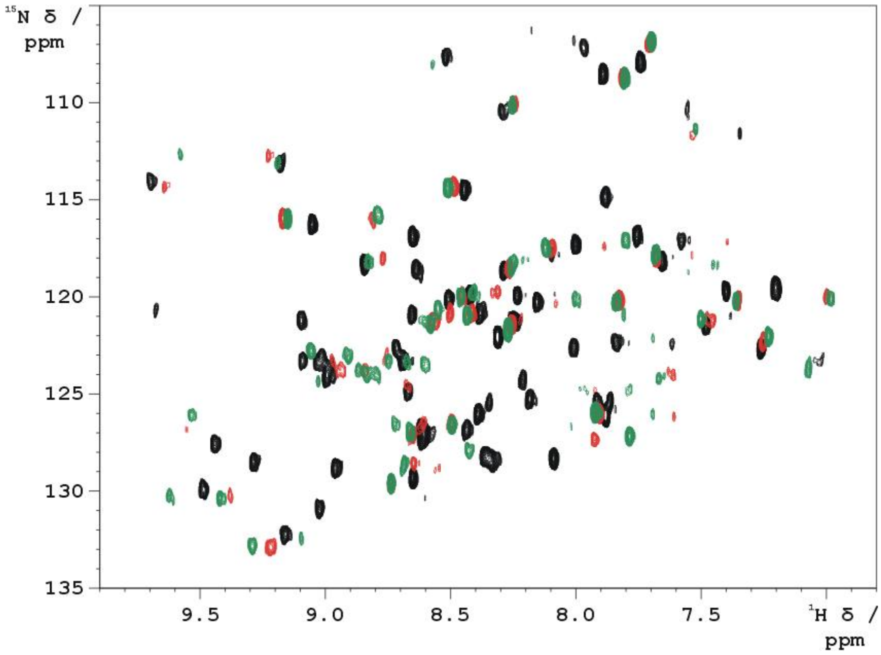

B

Supplementary Figure 5: ^{1}H-^{15}N TROSY spectra of RsmN, R62A mutant and bound complexes with RNAs

(A) Overlayed 800 MHz ^{1}H-^{15}N HSQC spectra of the wt-RsmN-His6 dimer (black) and RsmN R62A-His$_6$ mutant (red) at 25 °C in 25 mM potassium phosphate pH 7, 50 mM NaCl.

(B) Overlaid 800 MHz ^{1}H-^{15}N TROSY spectra of *P. aeruginosa* RsmN-His$_6$ in the absence of RNA (black), the presence of the RBS of gene PA1003 / *pqsR* (red) and the presence of RsmZ hairpin 2 (green).

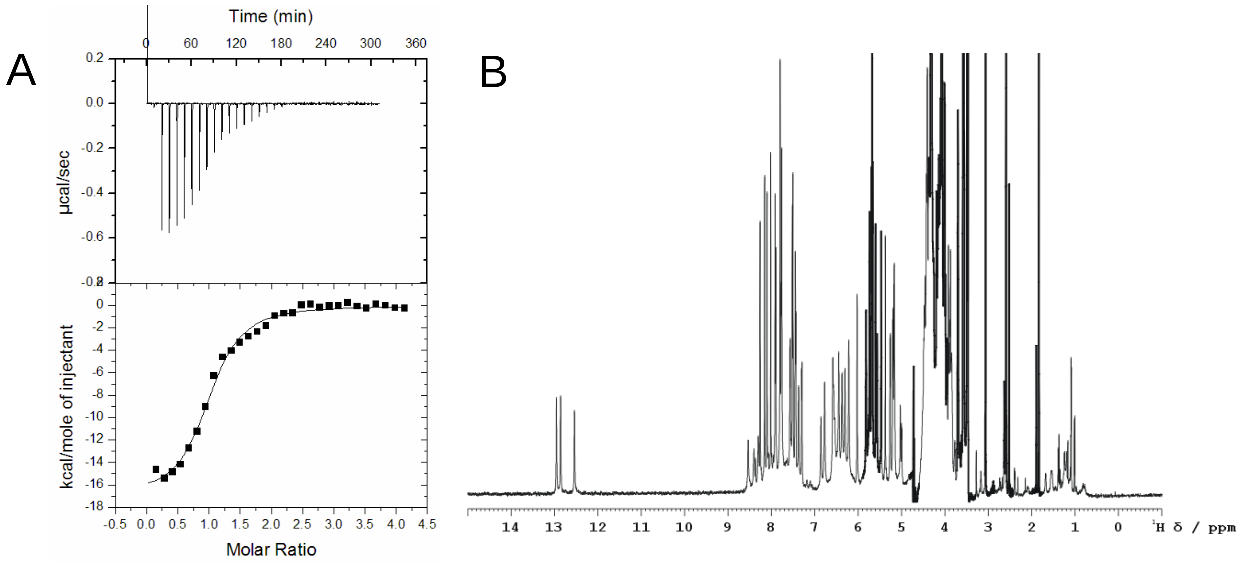

Supplementary Figure 6: ITC and NMR analysis of RsmY-1 and its RsmN complex

(A) ITC analysis of the binding of RsmY hairpin 1 to the RsmN dimer at 298K (K_d = 526 ± 65 nM). RNA (125 µM RNA, 25 mM potassium phosphate buffer pH 7, 50 mM NaCl) was titrated into a cell containing 1.424 mL protein (5□10 µM protein, 25 mM potassium phosphate buffer pH 7, 50 mM NaCl).

(B) 800 MHz ^{1}H NMR of RsmY hairpin 1 (RsmY-1) at 298K, phosphate buffered at pH 7.0, showing imino proton resonances in the 12.5 to 13 ppm region, consistent with hydrogen bonded Watson-Crick base pairing in a hairpin motif.

Supplementary Table 1: Source of the CsrA, RsmA, RsmE and RsmN homologues used for the phylogenetic analysis (Fig. 7)

Abbreviation	Organism	NCBI Accession Number
**Buchnera**	*Buchnera aphidicola* Sg	NP_660732
**Escherichia**	*Escherichia coli* str. K12, substr. W3110	NP_417176
**P. aeruginosa B136**	*Pseudomonas aeruginosa* B136-33	YP_007710694
**P. aeruginosa**	*Pseudomonas aeruginosa* PAO1	NP_249596 (RsmA),<br>YP_793655 (RsmN) (1)
**P. fluorescens Pf0**	*Pseudomonas fluorescens* Pf0-1	YP_350001 (RsmA),<br>YP_347644 (RsmE)
**P. fluorescens SBW25**	*Pseudomonas fluorescens* SBW25	YP_002874264 (RsmA),<br>YP_002873715 (RsmE)
**P. fulva**	*Pseudomonas fulva* 12-X	YP_004474215
**P. mendocina DLHK**	*Pseudomonas mendocina* DLHK	WP_003247014
**P. mendocina NK01**	*Pseudomonas mendocina* NK-01	YP_004380571
**P. mendocina ymp**	*Pseudomonas mendocina* ymp	CP000680.1|4335168-4335389(2)
**P. protegens RsmA**	*Pseudomonas protegens* (ex-*fluorescens*) CHA0	AAD33682 (RsmA),<br>AAT27429 (RsmE)
**P. pseudoalcaligenes**	*Pseudomonas pseudoalcaligenes* KF707	WP_003449739
**P. psychrotolerans**	*Pseudomonas psychrotolerans* L19	WP_007162777
**P. putida KT2440**	*Pseudomonas putida* KT2440	NP_746583 (RsmA),<br>NP_745962 (RsmE)
**P. stutzeri DSM10701**	*Pseudomonas stutzeri* DSM10701	YP_006524540
**P. stutzeri NF13**	*Pseudomonas stutzeri* NF13	WP_003298085
**P. syringae**	*Pseudomonas syringae* pv. tomato str. DC3000	NP_791668 (RsmA),<br>NP_793345 (RsmE)
**Pectobacterium**	*Pectobacterium carotovorum* subsp. *carotovorum* 71	P0DKY7
**Photorhabdus**	*Photorhabdus luminescens* subsp. *laumondii* TTO1	NP_928562
**pMBA19a**	plasmid pMBA19a, found in *Sinorhizobium meliloti*	AAX19276
**Proteus**	*Proteus mirabilis* HI4320	CAR40967
**Salmonella**	*Salmonella enterica* subsp. *enterica* ATCC9150	NP_806428
**Serratia**	*Serratia marcescens* CH-1	AAC25783
**Shewanella**	*Shewanella oneidensis* MR-1	AAN56423
**Vibrio**	*Vibrio cholerae* O1 biovar El Tor str. N16961	AAF93716
**Xanthomonas**	*Xanthomonas campestris* pv. *campestris* ATCC33913	YP_243576
**Xylella**	*Xylella fastidiosa* 9a5c	AAF82938|AE003866
**Yersinia**	*Yersinia enterocolitica* subsp. *enterocolitica* 8081	YP_001005173

(1)RsmN sequence of strain PA14, which is identical to that of PAO1.
(2)Un-annotated ORF, hence the corresponding nucleotide sequence is given.

SUPPLEMENTAL EXPERIMENTAL PROCEDURES

Bacterial growth and maintenance

The strains and plasmids used in this study are listed in Table 1. Both *E. coli* and *P. aeruginosa* strains were routinely grown in LB broth or LB agar plates 37°C. Where required, antibiotics were added to media at the following concentrations: tetracycline, 25 µg/ml (*E. coli*) or 125 µg/ml (*P. aeruginosa*); gentamicin, 10 µg/ml; chloramphenicol, 250 µg/ml; spectinomycin, 1,000 µg/ml (*P. aeruginosa*). To counterselect *E. coli* S17-1 donor cells in matings with *P. aeruginosa*, nalidixic acid was used at a concentration of 10 µg/ml. Enrichment for tetracycline-sensitive clones was performed with tetracycline (20 µg/ml) and carbenicillin (2,000 µg/ml) as bacteriostatic and bactericidal antibiotics, respectively.

P. aeruginosa DNA manipulation

To screen for genes capable of restoring swarming in a *P. aeruginosa rsmA* mutant**,** genomic DNA was prepared from *P. aeruginosa* as described previously (Gamper et al., 1992), partially digested by *Sau*3AI and 2-4 kb fragments cloned into pME6000, The resulting plasmids were transformed into the *P. aeruginosa rsmA* mutant strain PAZH13 and each clone screened for restoration of swarming. To reduce the size of the 2.5-kb chromosomal fragment cloned in pPAMMB-16, the 1.7-kb *Pst*I-(*Sph*I)$_{\text{T4 DNA Pol.}}$ fragment was first subcloned in pME6000 digested with *Pst*I and (*Bam*HI)$_{\text{T4 DNA Pol.}}$ and the 1.25-kb *Pst*I-*Nhe*I fragment was deleted from the resulting plasmid. The resulting plasmid, pHS2, has an insert corresponding to the 0.47-kb *Nhe*I-*Sph*I chromosomal fragment that carries *rsmN*. The *rsmN*R62A allele was obtained commercially (Integrated DNA Technologies, IDT) as a 0.49-kb *Eco*RI-*Spe*I fragment equivalent to the insert in pHS2 and inserted in pME6000 to generate pHS2R62A (Table 1). Plasmid pMM33, for the in-frame deletion of *rsmN,* was constructed by joining in the suicide plasmid pDM4 0.54-kb upstream and downstream fragments obtained by PCR amplification of chromosomal DNA with, respectively, oligonucleotides RSMNU and RSMND (Table 1). Suicide plasmid construction and allelic replacement in *P. aeruginosa* was carried out as described elsewhere (Fletcher et al., 2007).

Protein production and RNA preparation

The pET-28b(+) expression system (Novagen) was used to express His-tagged RsmA (His_6-Thb-RsmA) and RsmN (His_6-Thb-RsmN proteins) within host *E. coli* C41(DE3) cells, where 'Thb' indicates an LVPRGS thrombin recognition sequence. His_6-fusion proteins were purified by using HisPur Cobalt Resin (Thermo Scientific). The manufacturer's

procedure using a Gravity-flow column was followed, except for the use of a different equilibration/wash buffer (50 mM K_2HPO_4, 500 mM NaCl, pH 7.5), an additional high salt wash step (50 mM K_2HPO_4, 1 M NaCl, pH 7.5) and a different elution buffer (50 mM K_2HPO_4, 500 mM NaCl, 1 M imidazole). Protein overexpression with incorporation of ^{13}C and ^{15}N labelling for NMR studies was performed in M9 minimal medium supplemented with ammonium chloride (1 g L^{-1} of $^{15}NH_4Cl$ and glucose (2 g L^{-1} $^{13}C_6H_{12}O_6$).

His-tagged proteins were further purified by gel filtration using a HiLoad 26/600 Superdex 75 pg column (GE Healthcare). Purified proteins were desalted into 25 mM NH_4OAc using a HiTrap Desalting column (GE Healthcare) and subsequently lyophilised. Identity and purity were verified by electrospray ionisation-mass spectrometry and SDS-PAGE. Multimeric state was confirmed by analytical gel filtration using a Superdex 75 column (Pharmacia Biotech), which was calibrated using a Gel Filtration LMW Calibration Kit (GE Healthcare).

Short RNA oligonucleotides were purchased from Dharmacon (Thermo Scientific), deprotected according to manufacturer's instructions, lyophilised and stored at -20°C. RNA stock solutions (~1 mM) were prepared from the lyophilised stocks. RNA hairpin formation was induced by thermally unfolding the RNA molecules at 95 °C for 1 min, with subsequent cooling and re-annealing monitored by NMR.

Analytical size exclusion chromatography (SEC)

Analytical SEC was used to confirm multimeric state of the protein after purification, as well as to monitor binding between protein and RNA. A Superdex 75 HR 10/30 analytical column (GE Life Sciences) was calibrated using a Gel Filtration LMW Calibration Kit (GE Life Sciences), which contained: aprotinin (6.5 kDa), ribonuclease A (13.7 kDa), carbonic anhydrase (29 kDa), ovalbumin (43 kDa), conalbumin (75 kDa) and blue dextran 2,000 (2 MDa). Absorbance at 280 nm was monitored to determine the elution volumes of injected samples and apparent molecular weights of species eluted in subsequent analytical SEC experiments were calculated. 50 µM protein samples in 25 mM potassium phosphate buffer pH 7, 150 mM NaCl were used to assess dimer formation of the purified RsmA and RsmN proteins. 50 µM protein and 100 µM RNA samples were used in RNA-binding SEC experiments, in a buffer containing 25 mM potassium phosphate pH 7, 50 mM NaCl.

Isothermal titration calorimetry

Isothermal titration calorimetry (ITC) experiments were recorded on a VP-ITC high sensitivity titration calorimeter (MicroCal) at 298 K. RNA and protein samples were degassed at 298 K for 10 min prior to the titration experiments. RNA (125 µM RNA, 25 mM potassium phosphate buffer pH 7, 50 mM NaCl) was titrated into a cell containing 1.424 mL protein (5□10 µM protein, 25 mM potassium phosphate buffer pH 7, 50 mM NaCl). Titrations consisted of one preliminary injection of 2 µL, followed by 29 injections of 10 µL, with 10 min intervals between injections. A constant stirring speed of 300 rpm ensured rapid mixing during the titration. A reference power of 6 µCal sec^{-1} was used. Data were analysed and fitted to a single-site model using Origin software (MicroCal).

NMR spectroscopy

NMR experiments were carried out on a Bruker Avance III 800 MHz spectrometer using an inverse detection QCI CryoProbe, fitted with Z-axis gradient. All spectra were acquired using standard Bruker pulse sequences. Protein samples in 600 µL were prepared in 25 mM potassium phosphate buffer pH 7.0, 150 mM NaCl in 90% H_2O/10% D_2O to give RsmA and RsmN protein sample concentrations of 100-200 µM. Protein-RNA complexes were prepared in the same buffer solutions (but reduced salt, 50 mM NaCl) by titrating 50 µM protein samples with RNA (5-100 µM). 1D ^{1}H NMR spectra were collected using an excitation sculpting water suppression pulse sequence over a 13 ppm spectral width (protein) and 22 ppm width (RNA). ^{13}C/^{15}N-labelled proteins were used to collect 2D [^{1}H,^{15}N]-transverse relaxation optimised spectroscopy (TROSY) experiments for structural comparisons. Although a series of HNCO, HNCA, HN(CO)CA, HN(CA)CO, CBCANH and CBCA(CO)NH triple resonance experiments were collected for assignment purposes the quality of the spectra was low and yielded only ca. 50% of the expected correlation peaks precluding a complete backbone assignment. NMR data were acquired and processed using the TopSpin software package (Bruker) and analysed with CCPNMR software.

Crystallisation and data collection

Crystals of His-tagged RsmN were grown from the PEGs screen (QIAGEN) using sitting drop vapour diffusion from equal 1μl volume mixtures of protein (500 µM H_6-RsmN, 10 mM Tris-HCl, 150 mM NaCl, pH 7.0) and reservoir solution (0.2 M NaI, 20% (v/v) PEG 3350). The crystals were indexed in the trigonal space group $P3_112$ and data collected to 2.0 Å resolution using the ID14-1 beamline at the ESRF (κ = 0.9334 Å). Data were processed and scaled using MOSFLM with a final R_{merge} of 0.07 (Table 2).

The RsmN complex with RsmZ-2 was isolated and concentrated to 50 µM. Initial crystallisation conditions were identified using the protein-complex suite (QIAGEN). These were grown by sitting drop vapour diffusion from equal 1 µl volume mixtures of protein (500 µM H_6-RsmN, 10 mM Tris-HCl, 150 mM NaCl, pH 7.0) and reservoir solution of 0.1 M $Mg(OAc)_2$, 0.1 M NaOAc pH 4.5 and 8% (w/v) PEG 8000. To increase the crystal size optimisation was carried out using 96 additives from the Hampton research screen. Seven additives were observed to increase the dimensions of the crystals grown. These were KCl (0.1 M), LiCl (0.1 M), 6-aminohexanoic acid (3% (w/v)), glycyl-glycyl-glycine (0.03 M), trimethylamine hydrochloride (0.01 M), dimethylbenzyl-ammonium propane sulfonate (or 'non-detergent sulphobetaine 256' (NDSB-256), 0.1 M) and benzamidine hydrochloride (2% (w/v)). The benzamidine hydrochloride additive was subsequently optimised further by varying the concentration between 1-4% and the largest crystals were grown from 3-4% (w/v) benzamidine hydrochloride, 0.08 M $Mg(OAc)_2$, 0.1 M NaOAc pH 4.5 and 4% (w/v) PEG 8000. Data were collected at DIAMOND beamline IO3 and processed to 3.2 Å resolution of using MOSFLM (Table 2).

Structure determination and refinement

The RsmN structure was solved by molecular replacement with PHASER (CCP4 suite) using the *P. aeruginosa* RsmA crystal structure (PDB code 1VPZ) as the search model. Molecular replacement identified one clear solution, corresponding to the highest peak in the cross-rotation function gave the correct orientation for a single molecule in the asymmetric unit and an initial R_{cryst} of 0.39. The resulting $2F_o$-F_c electron density map was of high quality with additional protein features observable outside of the boundary of the search model and in the F_o-F_c difference electron density map. Extensive model rebuilding using composite omit electron maps (phenix) was required to model the α-helical 16-residue insertion in RsmN that is absent in RsmA. Refinement was performed using REFMAC5, with model building carried out using Coot. The model was refined to R_{cryst} and R_{free} values of 0.24 and 0.29, respectively. The final model includes RsmN residues 1-66, residues Gly-Ser from the N-terminal thrombin cleavage site, 2 iodide ions and 42 water molecules. 95.6% of residues lie in the most favoured region of the Ramanchandran plot, with the remainder lying in the additional allowed area (Table 2).

The structure of the RsmN-RNA complex was determined using molecular replacement with PHASER (CCP4 suite) with the RsmN structure as a model identifying two molecules in the asymmetric unit. This resulted in high quality electron density for the protein with sufficient space and additional electron density present in Fo-Fc difference maps surrounding the

dimer suggestive of the presence of two RNA hairpins in the asymmetric unit. Model building of the RNA was carried out using Coot and refinement was performed using REFMAC5 with NCS restraints in place. The model was refined to R_{factor} and R_{free} values of 0.22 and 0.32, respectively (Table 2). Analysis of the stereochemical quality of the structure was carried out using MolProbity (Chen et al., 2010).

REFERENCES FOR SUPPLEMENTAL EXPERIMENTAL PROCEDURES

Chen, V.B., Arendall, W.B.r., Headd, J.J., Keedy, D.A., Immormino, R.M., Kapral, G.J., Murray, L.W., Richardson, J.S., and Richardson, D.C. (2010). MolProbity: all-atom structure validation for macromolecular crystallography. Acta Crystallogr D Biol Crystallogr *66*, 12-21.

Fletcher, M.P., Diggle, S.P., Crusz, S.A., Chhabra, S.R., Camara, M., and Williams, P. (2007). A dual biosensor for 2-alkyl-4-quinolone quorum-sensing signal molecules. Environ Microbiol *9*, 2683-2693.

Gamper, M., Ganter, B., Polito, M.R., and Haas, D. (1992). RNA processing modulates the expression of the *arcDABC* operon in *Pseudomonas aeruginosa*. J Mol Biol *226*, 943-957.

Heeb, S., Kuehne, S.A., Bycroft, M., Crivii, S., Allen, M.D., Haas, D., Cámara, M., and Williams, P. (2006). Functional analysis of the post-transcriptional regulator RsmA reveals a novel RNA-binding site. J Mol Biol *355*, 1026-1036.

Henrick, K., and Thornton, J.M. (1998). PQS: a protein quaternary structure file server. Trends Biochem Sci *23*, 358-361.

Heurlier, K., Williams, F., Heeb, S., Dormond, C., Pessi, G., Singer, D., Cámara, M., Williams, P., and Haas, D. (2004). Positive control of swarming and lipase production by the post-transcriptional RsmA/RsmZ system in *Pseudomonas aeruginosa* PAO1. J Bacteriol *186*, 2936-2945.

Romeo, T., Gong, M., Liu, M.Y., and Brun-Zinkernagel, A.M. (1993). Identification and molecular characterization of *csrA*, a pleiotropic gene from *Escherichia coli* that affects glycogen biosynthesis, gluconeogenesis, cell size, and surface properties. J Bacteriol *175*, 4744-4755.

Schrodinger, LLC (2010). The PyMOL Molecular Graphics System, Version 1.3r1.
